# Increasing Awareness for Peripheral Artery Disease through the Identification of Novel Biomarkers

**DOI:** 10.3390/biom13081189

**Published:** 2023-07-30

**Authors:** Ben Li, Muzammil H. Syed, Mohammad Qadura

**Affiliations:** 1 Division of Vascular Surgery, St. Michael’s Hospital, Unity Health Toronto, University of Toronto, Toronto, ON M5B 1W8, Canada; benx.li@mail.utoronto.ca (B.L.); muzammil.syed@mail.utoronto.ca (M.H.S.); 2 Department of Surgery, University of Toronto, Toronto, ON M5S 1A1, Canada; 3 Keenan Research Centre for Biomedical Science, Li Ka Shing Knowledge Institute, St. Michael’s Hospital, Unity Health Toronto, University of Toronto, Toronto, ON M5B 1W8, Canada

Peripheral artery disease (PAD) is a chronic atherosclerotic disorder that involves the lower extremity arteries, manifesting in claudication, rest pain, and tissue loss [1]. Affecting over 200 million people worldwide, PAD is a major contributor to decreased quality of life, limb loss, and death [2,3,4]. Referred to as a global pandemic, PAD disproportionally affects those in low- and middle-income countries [5]. Despite increasing evidence demonstrating the significant negative impact of PAD on patient lives and healthcare costs [6,7,8], it remains underdiagnosed and undertreated [9,10,11,12,13]. This is largely due to a lack of awareness of PAD by both patients and clinicians [14,15,16]. In 2021, Bauersachs and colleagues published an international survey including 9000 respondents from North America, South America, and Europe, showing that most people were not familiar with PAD or its health consequences [17]. Similarly, a survey of vascular surgeons in Canada demonstrated poor knowledge of recommended treatment targets, inadequate evaluation of risk factors, and suboptimal initiation of medications [18]. Clearly, the lack of awareness regarding PAD is a serious problem that requires further attention. 

Unlike conditions that have high rates of public and provider awareness, such as heart and kidney disease, no routinely available diagnostic or prognostic biomarker exists for PAD [19,20]. For heart disease, troponin and brain natriuretic peptides are regularly used to identify patients with myocardial infarction and heart failure, respectively [21,22]. Similarly, creatinine is routinely measured to assess kidney function [23]. These blood-based investigations are valuable because they are relatively inexpensive, accessible, easily collected, assessed, and interpreted, and multiple tests can be run in parallel, which is critical in busy clinical settings [24]. As a result, and among other reasons, the awareness and subsequent management of patients with heart and kidney disease is significantly better than for PAD [25,26]. Unfortunately, accessible blood-based tests for PAD do not currently exist in the clinical setting, leading to delayed diagnosis and suboptimal management [27].

Currently, the gold standard for PAD screening is the ankle–brachial index (ABI) [28]. This test generally requires referral to a vascular laboratory with trained vascular technicians, who use blood pressure cuffs to measure the systolic pressure at the ankles divided by the highest systolic brachial pressure [28]. This returns a ratio that helps a clinician understand relative blood flow in the lower extremities [28]. However, the ABI is limited by operator dependence, erroneous interpretation, relatively high cost (approximately USD 100 per test), and unreliability in patients with diabetes due to calcified vessels [29,30,31]. Furthermore, ABIs are rarely performed in the primary care setting as generalists’ lack of comfort with ordering and interpreting this test [32]. A 2021 survey found that 79% of primary care providers do not perform ABIs routinely in their clinical practice, citing time constraints, unavailability of skilled personnel, and complexity of result interpretation as major barriers [33]. Most clinicians view alternative forms of diagnosis, such as a blood or urine test, as preferable to ABIs and would enhance diagnostic efficiency [33]. Therefore, the introduction of an accessible and relatively inexpensive biomarker for PAD may overcome some of the challenges faced when using the ABI as a screening method for PAD, thereby improving the diagnosis and management of patients with PAD. Ultimately, our group believes that the introduction of an accessible blood-based biomarker for PAD may help contribute to increased awareness of this condition by providers and patients.

Several traditional biomarkers have been studied for PAD, including C-reactive protein (CRP), erythrocyte sedimentation rate (ESR), and interleukins (IL) 1/6 [34,35]. Unfortunately, these have not translated to routine clinical practice partly because of non-specificity to PAD, as they are also elevated in patients with other cardiovascular conditions such as coronary or cerebrovascular disease [36,37]. Recently, several biomarkers specific to PAD have been identified in the research setting, including fatty acid binding protein 3 (FABP3) [38,39,40,41]. This blood- and urine-based biomarker was demonstrated to be independently associated with the diagnosis of PAD and the prognosis of PAD-related adverse events, including major amputation and the need for surgical intervention [38,39,40,41]. The validation of these biomarkers for clinical implementation is ongoing and holds promise for improving PAD awareness.

In summary, PAD is a serious vascular condition that contributes to significant morbidity and mortality worldwide, particularly affecting marginalized populations [42,43]. It is often misdiagnosed and undertreated due to a lack of accessible tests, contributing to poor awareness of this condition by patients and providers. Investment into the development of novel biomarkers for PAD may provide an avenue to improve the diagnosis, management, and awareness of this condition, contributing to improved patient outcomes and reduced healthcare costs.
